# Novel Pactamycin Analogs Induce p53 Dependent Cell-Cycle Arrest at S-Phase in Human Head and Neck Squamous Cell Carcinoma (HNSCC) Cells

**DOI:** 10.1371/journal.pone.0125322

**Published:** 2015-05-04

**Authors:** Gunjan Guha, Wanli Lu, Shan Li, Xiaobo Liang, Molly F. Kulesz-Martin, Taifo Mahmud, Arup Kumar Indra, Gitali Ganguli-Indra

**Affiliations:** 1 Department of Pharmaceutical Sciences, College of Pharmacy, Oregon State University, Corvallis, Oregon, United States of America; 2 Molecular and Cell Biology Program, Oregon State University, Corvallis, Oregon, United States of America; 3 Department of Dermatology, Oregon Health and Science University, Portland, Oregon, United States of America; 4 Environmental Health Science Center, Oregon State University, Corvallis, Oregon, United States of America; Rush University Medical Center, UNITED STATES

## Abstract

Pactamycin, although putatively touted as a potent antitumor agent, has never been used as an anticancer drug due to its high cytotoxicity. In this study, we characterized the effects of two novel biosynthetically engineered analogs of pactamycin, de-6MSA-7-demethyl-7-deoxypactamycin (TM-025) and 7-demethyl-7-deoxypactamycin (TM-026), in head and neck squamous cell carcinoma (HNSCC) cell lines SCC25 and SCC104. Both TM-025 and TM-026 exert growth inhibitory effects on HNSCC cells by inhibiting cell proliferation. Interestingly, unlike their parent compound pactamycin, the analogs do not inhibit synthesis of nascent protein in a cell-based assay. Furthermore, they do not induce apoptosis or autophagy in a dose- or a time-dependent manner, but induce mild senescence in the tested cell lines. Cell cycle analysis demonstrated that both analogs significantly induce cell cycle arrest of the HNSCC cells at S-phase resulting in reduced accumulation of G_2_/M-phase cells. The pactamycin analogs induce expression of cell cycle regulatory proteins including master regulator p53, its downstream target p21^Cip1/WAF1^, p27^kip21^, p19, cyclin E, total and phospho Cdc2 (Tyr15) and Cdc25C. Besides, the analogs mildly reduce cyclin D1 expression without affecting expression of cyclin B, Cdk2 and Cdk4. Specific inhibition of p53 by pifithrin-α reduces the percentage of cells accumulated in S-phase, suggesting contribution of p53 to S-phase increase. Altogether, our results demonstrate that Pactamycin analogs TM-025 and TM-026 induce senescence and inhibit proliferation of HNSCC cells via accumulation in S-phase through possible contribution of p53. The two PCT analogs can be widely used as research tools for cell cycle inhibition studies in proliferating cancer cells with specific mechanisms of action.

## Introduction

Head and neck squamous cell carcinoma (HNSCC) is the sixth most common form of cancer in the world, accounting for 4% of all cancers in the United States [1, 2]. However, the lack of a significant increase in overall survival, cancer recurrence and unsuccessful treatment continues to crop up in a significant proportion of patients [3]. In spite of developing large number of synthetic drugs to target HNSCC [4–7], survival rates for HNSCC have remained fairly unaltered in the past three decades [8]. Synthetic drugs like cisplatin, carboplatin, methotrexate, 5-fluorouracil, paclitaxel, imatinib and cetuximab, in spite of showing variable degrees of efficacy, have been reported to cause deleterious side effects extensively [9–13]. Therefore, it is imperative to develop novel sustainable drugs that can be securely incorporated into current HNSCC treatment regimens, improving both tolerability and efficacy of treatment. In recent years, various natural products, used alone or in combination therapy, have demonstrated potential in cancer prevention [14, 15]. The anticancer effects of these compounds were achieved either *via* cell cycle arrest, apoptosis, and autophagy or by induction of senescence [16–19].

Pactamycin (PCT), a reputed natural product (isolated from *Streptomyces pactum*) that was first reported in the 1960s [20, 21], is a structurally unique antibiotic possessing strong antimicrobial [22], antitumor [23], anti-viral [24] and anti-protozoal [25] activities. Although PCT has been extensively studied for its antitumor potential, its use as an effective anticancer drug was compromised by its extensive cytotoxicity [21, 26]. For many years, efforts to obtain derivatives and analogs of PCT by standard synthetic organic chemistry approaches were hampered, mostly due to it complex chemical structure [21]. However, the recent total syntheses of PCT in sufficient quantities [26–29] and its synthetic derivatives [30] augur well for expanding the range of analogs for further testing. Using biosynthetic engineering approach, we have developed two novel PCT analogs, de-6MSA-7-demethyl-7-deoxypactamycin (TM-025) and 7-demethyl-7-deoxypactamycin (TM-026). We have demonstrated that both compounds are biologically active and nearly thirty times less toxic towards mammalian cells than their mother compound PCT [21]. The main aim of this study was to investigate the effects of TM-025 and TM-026 in HNSCC, and elucidate their mechanism(s) of action at the cellular and molecular level.

## Materials and Methods

### Reagents and kits

TM-025 and TM-026 were synthesized and characterized as described earlier [21]. CellTiter 96 Non-Radioactive MTT assay kit (#G4000) and DeadEnd Fluorometric TUNEL System kit (#G3250) were purchased from Promega Biosystems, CA. Click-iT protein Reaction Buffer Kit (#C10276) was obtained from Life Technologies, Inc., OR). Propidium iodide (1mg/ml) (#40017) was procured from Biotium, Inc., CA. Dnase I (RNase-free) (#79254) was obtained from Qiagen Inc., CA. RNase A (10mg/ml; DNase and protease-free) (#EN0531) was purchased from Fermentas Inc., MD. The remaining chemicals and solvents used were of standard analytical and HPLC grade. p53 inhibitor pifithrin-α hydrobromide (PFT) (#1267) was purchased from Tocris R&D Systems, MN. siRNA for Cdc2 (sc-29252) and control siRNA (sc-37007) were purchased from Santa Cruz Biotechnology Inc., CA.

### Antibodies

Rat monoclonal anti-BrdU (#OBT0030; AbDSerotec, Raleigh, NC), rabbit polyclonal anti-cytoskeletal actin (*β*-actin) (#A300-491A; Bethyl Laboratories, Inc., Montgomery, TX) and mouse monoclonal anti-Ki67 (NCL-Ki67-MM1; Novocastra/Leica Microsystems, Inc., New Castle, UK), rabbit polyclonal to p85 fragment of PARP (#G734A, Promega, Madison, WI) and cyclin B (BD #61029; BD Biosciences, San Jose, CA) were procured from their respective manufacturers. Rabbit polyclonal to cleaved caspase-3 (#9661), p21^Cip1/WAF1^ (#CS2947), Cdc25C (#CS4648), p-Cdc25C (#CS9528), Cdc2 (#CS9112) and p-Cdc2 (#CS9111) were purchased from Cell Signaling Technology, Inc., Danvers, MA. Antibodies for p53 (#SC-6243), p27^kip21^ (#SC528), p19 (#SC-71810), cyclin E (#SC481), Cdk2 (#SC6248) and Cdk4 (#SC23896) were all from Santa Cruz Biotechnology Inc., CA. Peroxidase-conjugated secondary (H&L chain-specific) antibodies for Western blots, goat anti-mouse IgG (#401253) and goat anti-rabbit IgG (#401315) were purchased from Calbiochem-Novabiochem Corp., CA. Secondary antibodies for immunocytochemical analyses, Cy3-AffiniPure donkey anti-rat IgG (#712-165-153) and Cy3-AffiniPure goat anti-mouse IgG (#115-165-003) were procured from Jackson Immuno Research Laboratories, Inc., West Grove, PA.

### Cell lines and culture

SCC25 (#30–2006; squamous cell carcinoma) and HPEK (human primary epidermal keratinocytes; #PCS-200-011) cell lines were obtained from ATCC, VA and SCC 104 cell line was kindly provided by Dr.Sussane M Gollins, University of Pittsburg through material transfer agreement [31]. SCC104 and SCC25 cells were grown in monolayer culture in DMEM/F12 medium supplemented with 10% fetal bovine serum (FBS) (Gibco-Invitrogen Corp., CA), antibiotics and hydrocortisone. HPEK cells were grown in dermal cell basal medium (PCS-200-030) containing 0.4% bovine pituitary extract, rhTGF-α, L-glutamine, hydrocortisone hemisuccinate, rh-insulin, epinephrine and apo-transferrin. All cells were grown in polystyrene-coated culture flasks in a humidified atmosphere with 5% CO_2_ at 37°C for all experiments, as well as for maintenance.

### MTT assay

The SCC25, SCC104 and HPEK cells (1.8 × 10^4^/ well in 200 μl culture medium in a 96-well plate) were treated with different doses (1, 10, 100, 500, 1000 and 5000 nM) of TM-025 and TM-026 for 24, 48 and 72 h. Percentage viability of cells was evaluated by the MTT assay, a putative test that evaluates formazan formation [32,33], for computation of mitochondrial succinate dehydrogenase activity as an estimate of live cells [34]. The experiment was performed using the Cell Titer 96 Non-Radioactive MTT assay kit (Promega Biosystems, CA), according to the manufacturer’s instructions. Cells treated with DMSO (1 μl per 200 μl medium) were considered as control groups (vehicle). End-point absorbance was determined at 570 nm (with a 650 nm reference) using a Synergy HT Multi-Mode Microplate Reader (BioTek U.S., VT). The percentage viability of cells were calculated estimated using the following formula: % viability = (A_***T***_/ A_***V***_) × 100, where A_***V***_ is the absorbance of the vehicle-treated control group and A_***T***_ is the absorbance of the test samples (TM-025/TM-026-treated).

### Detection of nascent protein synthesis

Click-iT protein Reaction Buffer Kit (#C10276; Life Technologies, Inc., OR) was used to detect synthesis of nascent protein, using manufacturer’s instructions. Briefly, SCC104 cells were grown in methionine-free medium for 1 h at 37°C, followed by the addition of 50 μM Click-iT AHA reagent. Simultaneously, TM-025/TM-026/PCT was added and treated for 30 and 90 min. Specific lysis buffer, as per the instruction manual, was used for preparing the protein lysates. Equal amount of proteins (40 μg) obtained after vehicle or PCT analog treatment, were incubated with Click-iT reaction buffers for different time point. The extent of biotinylation was measured by Western blot, using a streptavidin-HRP conjugate and chemiluminescent substrate.

### Immunocytochemistry (ICC) analyses

Cells were seeded (2 × 10^5^) in each well of 6-well plates and allowed to adhere for 24 h. The cells were then treated with 0, 1, 5, 10, 20 or 50 nM of TM-025 or TM-026 (in culture medium) for 48 h, after which they were fixed with acetone-methanol (50:50). Fixed cells were stained for proliferation marker Ki67 using anti-Ki67 antibody and labeled with Cy3-tagged secondary antibody for visualization under a fluorescence microscope (Zeiss Axio Imager Z1, Carl Zeiss, NY). DAPI was used as a counter-stain. Cells were quantified using the ImageJ software (NIH, USA). Furthermore, for the time-kinetics assay, similar treatments were carried out for 24 h in the squamous carcinoma cells. In order to label S-phase cells, bromodeoxyuridine (BrdU) (10 μM final concentration in culture medium) pulse labeling was performed on cells (SCC25, SCC104 and HPEK) treated with 0, 1, 10 and 50 nM of TM-025 or TM-026 for 48 h, fixed and analyzed by ICC [35]. An anti-BrdU antibody was used to detect BrdU incorporation, indicating cells that were actively replicating their DNA [36].

### Cell cycle analysis by flow cytometry

Cell cycle analysis was performed by flow cytometry, following a previously described protocol [37], with a few modifications. In brief, SCC25 and SCC104 cells were treated with 1, 10 and 50 nM of each of the PCT analogs for 24 h. The cells were then fixed with ice-cold 70% ethanol (overnight at -20°C), permeabilized with 0.1% Triton X-100 (in PBS), treated with RNase A and stained with propidium iodide. The DNA content was analyzed with a Flow Cytometer (Beckman Coulter, Inc., CA). The cell cycles were analyzed using MultiCycle for Windows (Phoenix Flow Systems, AZ). DMSO (vehicle)-treated cells were considered as the control group. Simultaneously, time-kinetics (1 nM of TM-025/TM-026 for 24, 48 and 72 h) and a dose kinetics (0, 1, 10 and 50 nM of TM-025/TM-026 treatment for 24 h) studies were done to observe changes in the facets of cell cycle progression.

### p53 inhibitor (PFT) experiments

PFT (50 nM) was added to the cells with ~50% confluency for pre-incubation. TM-025 and TM-026 were added 24 h after addition of PFT, and incubated for 72h. Vehicle was used as control. PFT was continued to be added every day until the cells were harvested at 72 h (total PFT concentrations were therefore 200nM). In order to confirm the inhibition of p53, whole cell lysates were prepared and western blot analyses were performed for p53 expressions as described below. Cell cycle analysis by flow cytometry was performed to analyze the effect of PFT on S-phase arrest as described above under cell cycle analysis protocol.

### Small interfering RNA (siRNA) treatment

We have analyzed the role of Cdc2 in cell cycle arrest using Cdc2 si-RNA and the scrambled siRNA as control (si-Con). Transfections were performed by modifying the methods as described [38]. Briefly, SCC25 cells were seeded in petri dishes, and transfections were performed when they reached ~30–50% confluency. 100 nmol of si-Cdc2 and si-Con were used according to the manufacturer’s instructions and were harvested at 48 h for western blot analysis to check the efficiency of Cdc2 knock-down, and cell-cycle analysis to determine effects of knock-down on S-phase arrest.

### Apoptosis, PARP cleavage and caspase-3 activation assay

TUNEL assay was performed according to manufacturer's instructions, using the DeadEnd Fluorometric TUNEL System kit (Promega Biosystems, CA) to determine apoptosis [39] in SCC25, SCC104 and HPEK cells treated with 50 and 100 nM of TM-025 or TM-026 for 24 h. Activation of apoptosis by cleavage of PARP [40,41] and caspase-3 was also determined by western blot in cells treated with a high dose (1 μM) of TM-025 and TM-026 for a period of 72 h [42,43]. For the cleaved caspase-3 and PARP cleavage study, commercially available caspase-3 control cell extract (#9663; Cell Signaling Technology, MA) was used as a positive control.

### Autophagy analysis

Autophagy was determined by detecting levels of LC3-I and LC3-II cytosolic proteins by Western blot. The LC3-II:LC3-I ratio, determined and compared with that of rapamycin-treated cells (positive control), revealed whether the PCT analogs cause autophagy in cells [44,45]. The cells were treated with pepstatin A to protect the endogenous LC3 turnover from being destroyed by lysosomal hydrolases [17]. The proteins (LC3-I and LC3-II) were probed by an anti-LC3 monoclonal antibody, and detected protein bands were quantified using Multi Gauge v2.3 gel image analysis software (Fujifilm Corporation, Tokyo).

### Senescence assay

Senescence-associated *β*-galactosidase (SA-*β*-gal) activity, which is a known characteristic of senescent cells (not observed in pre-senescent, quiescent or immortal cells) [46], was estimated as described [47]. Briefly, sub-confluent cultures of SCC25 and SCC104 cell lines were maintained in 6-well plates and treated with 200 nM of TM-025 and TM-026 for 72 h. Following incubation, cells were fixed and subjected to SA-*β*-gal staining solution and the intracellular blue insoluble precipitate in the cell was visually observed [47]. Long-term treatment in SCC25 cell line was done with 500 nM of the PCT analogs for 4 days, followed by replenishing fresh medium with the compound in one set and only with fresh medium in another set. SA-*β*-gal staining was then performed in both the treatment groups mentioned above. Images were taken at 20X magnifications using Leica DME microscope (Leica Microsystems Inc., IL) fitted with Leica DFC280 digital camera (Leica Microsystems Imaging Solutions Ltd., Cambridge) and analyzed using Leica Application Suite v3.3.0 (Leica Microsystems Ltd., Switzerland) and Adobe Photoshop CS4 (Adobe Systems Inc., CA). These experiments were repeated three times.

### Western blot analyses

Western blot analyses were performed with lysates of SCC104 cells. Protein concentrations were measured using the BCA protein assay kit (Pierce Chemical Co., Rockford, IL). Cell lysates were electrophoresed in 10% SDS-PAGE and transferred onto nitrocellulose membrane (GE Water & Process Technologies, PA) by electro-blotting. Membranes were blocked with 5% non-fat milk (in 1% TBS-T buffer) for 30 min at room temperature and then probed with specific antibodies against the investigated proteins. Peroxidase-tagged secondary antibodies were employed to detect protein bands using the ECL Western blotting substrate (GE Water & Process Technologies, PA). Protein bands (that were detected on X-ray films) were scanned and their intensities were quantified using Multi Gauge v2.3 gel image analysis software (Fujifilm Corporation, Tokyo) and normalized using *β*-actin as control. The quantified value for each band was used to extrapolate the percentage change in expression of the protein (due to TM-025/TM-026 treatment) in a two-step normalization method:

- *Calculation of Relative Intensity (RI):*
  $$\text{RI}=\frac{\text{Intensity of protein band}}{\text{Intensity of the corresponding}\;\beta \text{-actin band}}$$
- *Calculation of percentage change in expression:*

$$\text{\% Change in expression =}\frac{\text{(RI of sample - RI of vehicle control)}}{\text{RI of vehicle control}}\times 100$$

### Statistical analyses

All analyses were carried out in triplicates. Data are presented as mean ± SEM (standard error of mean). Statistical analyses were performed by one-way ANOVA to determine significant differences between groups at p<0.05. Estimated correlations were also tested for significance (by two-tailed unpaired *t*-test) at the same confidence limit. MATLAB v.R2010 (Natick, MA), GraphPad Prism 5.00 (La Jolla, CA) and Microsoft Excel 2007 (Roselle, IL) were used for the statistical and graphical evaluations.

## Results

### TM-025 and TM-026 inhibit growth of HNSCC cells (SCC25 and SCC104)

**Fig 1:**
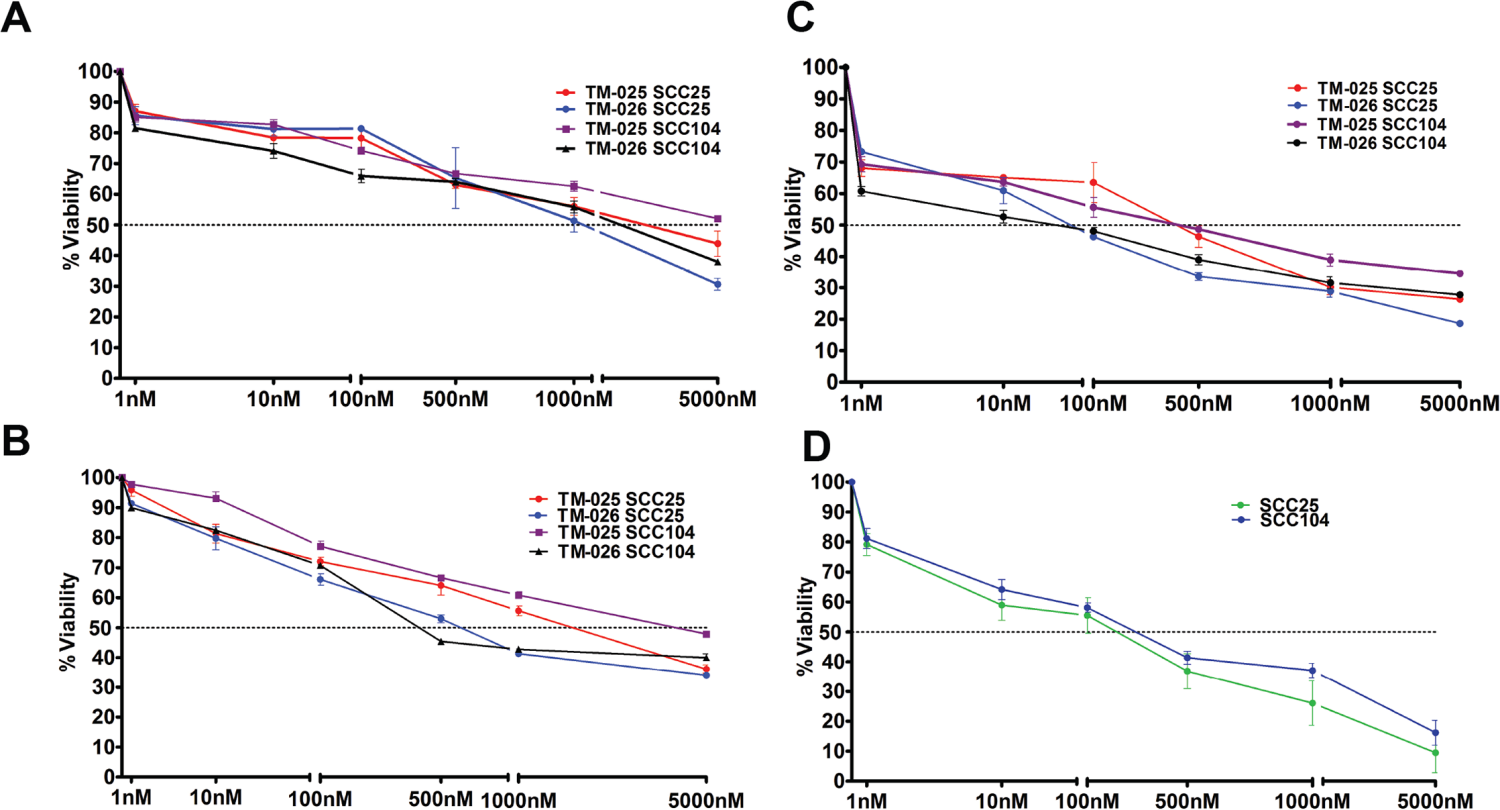
PCT analogs reduced viability of human head and neck cancer cells (SCC25 and SCC104) in a dose-dependent manner. (A-C): 1×10^4^ cells of each type were treated with TM-025 and TM-026 for 24, 48 and 72 h (A, B and C, respectively) in increasing concentrations (1, 10, 100, 500, 1000 and 5000 nM). Percentage viability of SCC104 and SCC25 cells (estimated by MTT assay) were significantly (P<0.05) reduced with higher doses of both analogs. (D): Effect of 48 h treatment of the parent compound PCT in SCC25 and SCC104 cell lines. All experiments have been performed in triplicates, and results obtained have been plotted as the data means (±SEM).

We hypothesized that PCT analogs TM-025 and TM-026 inhibit cellular growth and proliferation. Dose-dependent and time-dependent MTT assays were performed to determine the effects of the PCT analogs on the viability of HNSCC cells. Both squamous carcinoma cell lines (SCC25 and SCC104) demonstrated considerable dose-dependent reduction in their viabilities when treated with increasing doses (0 to 5000 nM) of TM-025 or TM-026 for 24, 48 and 72 h (Fig 1A, 1B and 1C). A significant time-dependent decline in the percentage of viable cells was detected within a time span of 24 to 72 h (Fig 1A–1C). IC_50_ for TM-025 on SCC25 cell line at 24 and 48 h time points were 3945.4 ± 17.23 nM and 2415.5 ± 23.12 nM, and for the SCC104 cell line were 5050.23 ± 32.13 nM and 4429.1 ± 19.54 nM, respectively. Similarly, IC_50_ for TM-026 in SCC25 at 24 and 48 h time points were 1071.2 ± 53.1 nM and 771 ± 47.2 nM, and for SCC104 were 3609.22 ± 44.62 nM and 423 ± 18 nM, respectively. However, IC_50_ of the parent compound PCT at 48 h is 93.61 ± 14.2 nM (Fig 1D), thereby suggesting a significantly (P<0.05) greater toxicity of PCT in comparison to its two analogs. Also, for 24 h treatment on primary human epidermal keratinocytes (HPEK), TM-025 and TM-026 showed IC_50_ values of 379 ± 44.2 nM and 253 ± 29.4 nM, respectively (S1 Fig). Altogether, our results indicate that pactamycin analogs TM-025 and TM-026 exhibit growth inhibitory effects in both HNSCC cell lines (SCC25 and SCC104) in a dose- and time-dependent manner.

### PCT analogs do not inhibit protein synthesis

**Fig 2:**
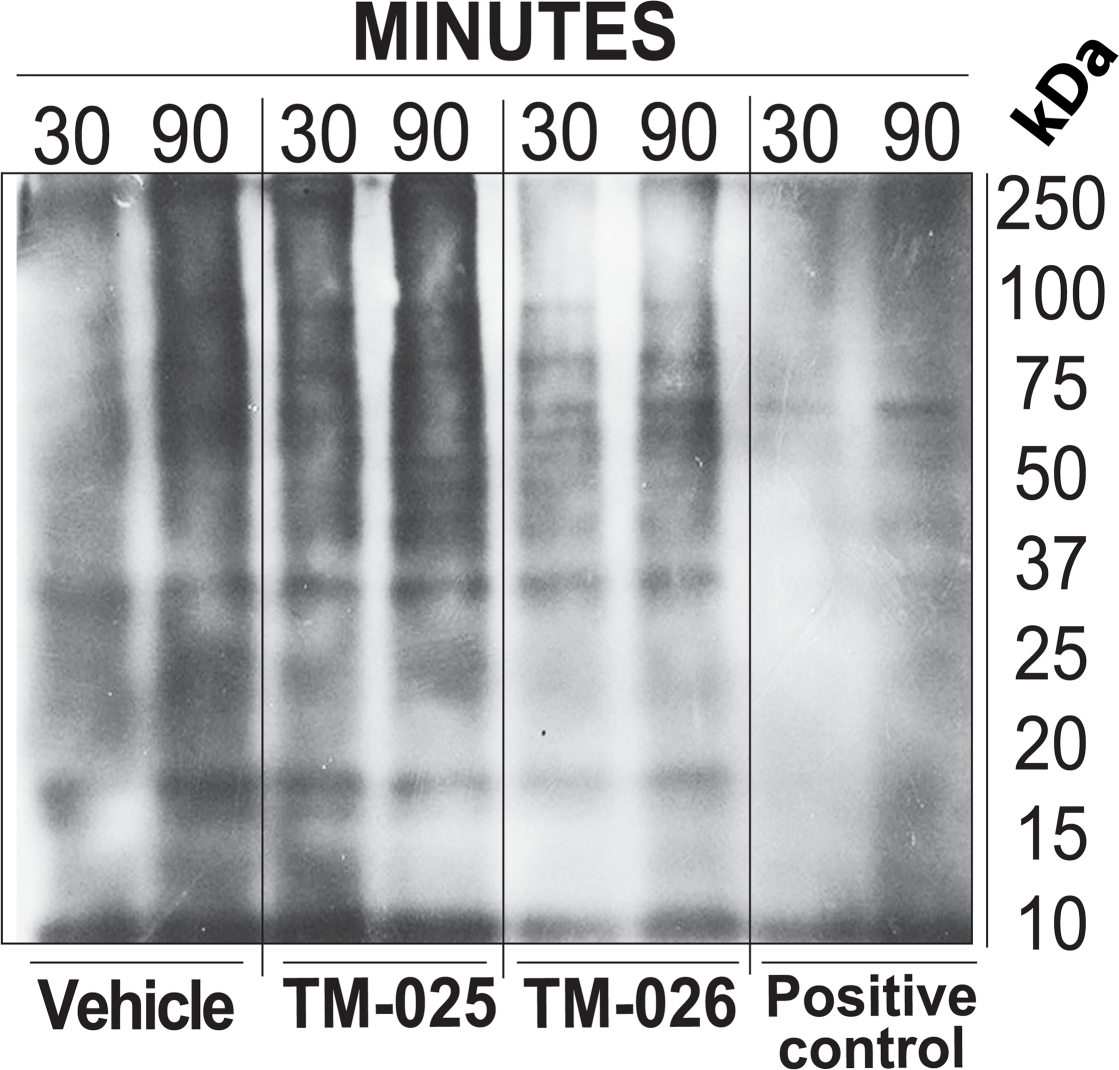
PCT analogs did not inhibit protein synthesis, which is a capstone property of PCT. Detection of protein synthesis was performed using the Click-iT azide-alkyne streptavidin HRP reaction model with Western blot. SSC104 cells were incubated in L-methionine-free medium containing 50 μM Click-iT AHA and subsequently treated with 100 nM of TM-025, TM-026 and PCT for 30 min and 90 min. Immunoblot shows the newly synthesized proteins (bands) in TM-025 and TM-026-treated cells, and a lack of such synthesis in PCT-treated cells.

We have previously observed that the PCT analogs were less cytotoxic and did not share some of the functional characteristics of the parent compound [21]. A general inhibition of protein synthesis is one of the reputed functions of PCT [20]. In order to test whether its analogs, TM-025 and TM-026, function similarly as a general inhibitor of protein synthesis, we analyzed their ability to inhibit synthesis of nascent protein using the Click-iT azide-alkyne streptavidin HRP reaction model, followed by Western blotting (for details see Methods). Immunoblot analyses revealed that, treatment with 100 nM of TM-025 and TM-026, could detect ladders of newly synthesized proteins of various molecular sizes, which were lacking when treated with the same amount of positive control and a known inhibitor of protein synthesis PCT (Fig 2). The above results indicate that PCT analogs TM-025 and TM-026 do not inhibit protein synthesis, unlike the parent compound PCT.

### TM-025 and TM-026 inhibit cell proliferation in a dose dependent manner

**Fig 3:**
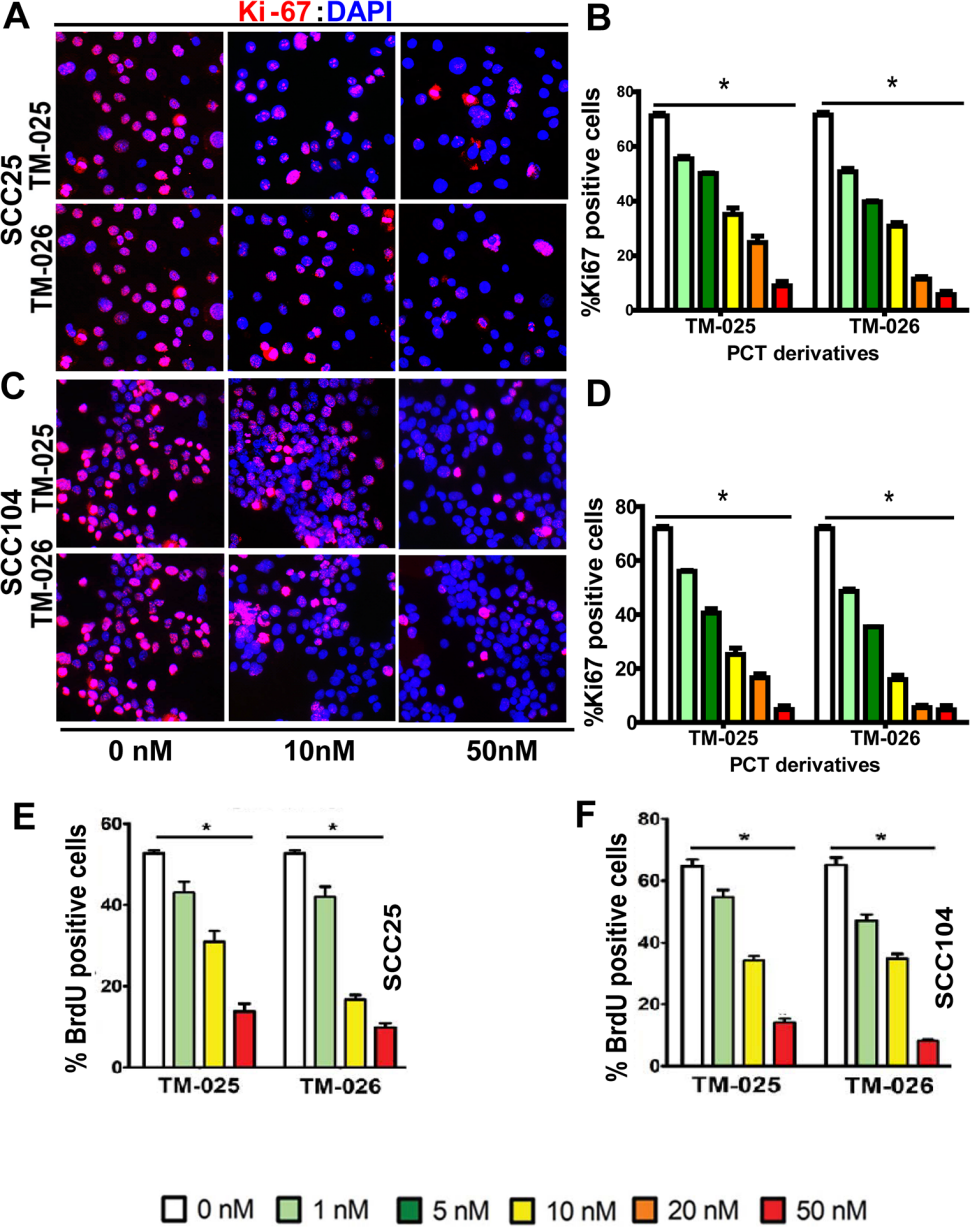
Dose-dependent inhibition of proliferation in SCC25 and SCC104 cells after treatment with PCT analogs. (A-D):48-h treatment with TM-025/TM-026 showed significant (P<0.0001; depicted with *) dose-dependent decrease in the levels of Ki67, a cell proliferation marker, in SCC25 (A and B) and SCC104 (C and D) cells. All experiments were performed in five replicates, and the results are presented as means (±SEM). (E-F): 48 h treatment with either of the PCT analogs exhibited a dose-dependent (0, 1, 10 and 50 nM) decrease in the percentage of actively proliferating (BrdU^+^) cells in SCC25 (E) and SCC104 (F) cells. Results were significant at P<0.0001 (*n* = 5), and depicted with *.

To investigate whether the two PCT analogs reduce cell viability by inhibiting cell proliferation, we examined their impact on the proliferation of the SCC25 and SCC104 cell lines. To that end, expression of Ki67, a mammalian nuclear protein that has been used as a marker of cell proliferation [48,49], was determined by an immunocytochemical approach using anti-Ki67 antibody after 48 h of treatment of SCC25 and SCC104 cells with each of the PCT analogs (for details, see Methods). A significant dose-dependent decrease was observed in proliferation rate in both the cell lines in the range of 1–50 nM for each of the two drugs tested (Fig 3A–3D). Post-48 h of treatment with 50 nM TM-025, the percentage of Ki67^+^ cells in SCC104 and SCC25 cell lines was significantly reduced to 4.811 ± 2.33 and 8.95 ± 2.75 (Fig 3A and 3B), respectively. Similarly after TM-026 treatment, the percentage of Ki67^+^ cells were reduced to 4.68 ± 2.69 and 5.74 ± 2.03 respectively (Fig 3C and 3D). Therefore, in congruity to the results of the MTT assay, both TM-025 and TM-026 inhibited 80–90% of cell proliferation at a concentration of 50 nM.

To confirm the results of Ki67 labeling, BrdU (a synthetic analog of thymidine) was used to label cells in the S-phase of the cell cycle [16, 50]. Post-pulse labeling of cells with BrdU, we determined BrdU incorporation into the newly synthesized DNA of replicating cells by immunocytochemistry using anti-BrdU antibody. Similar to the results obtained for Ki67 labeling, the percentage of BrdU^+^ cells was diminished with increasing doses of TM-025 and TM-026 in SCC25 (Fig 3E) and SCC104 (Fig 3F) cells after 48 h of treatment. Briefly, the percentages of BrdU^+^ cells at 48 h of treatment with 50 nM of TM-025 in SCC104 and SCC25 cell lines were reduced to 12.08 ± 1.32 and 9.13 ± 1.5 and for TM-026 were 9.87 ± 1.84 and 5.00 ± 2.15, respectively. The above results demonstrated that the two PCT analogs inhibit proliferation of HNSCC cell lines in a dose-dependent manner.

### PCT analogs do not induce apoptosis or autophagy

**Fig 4:**
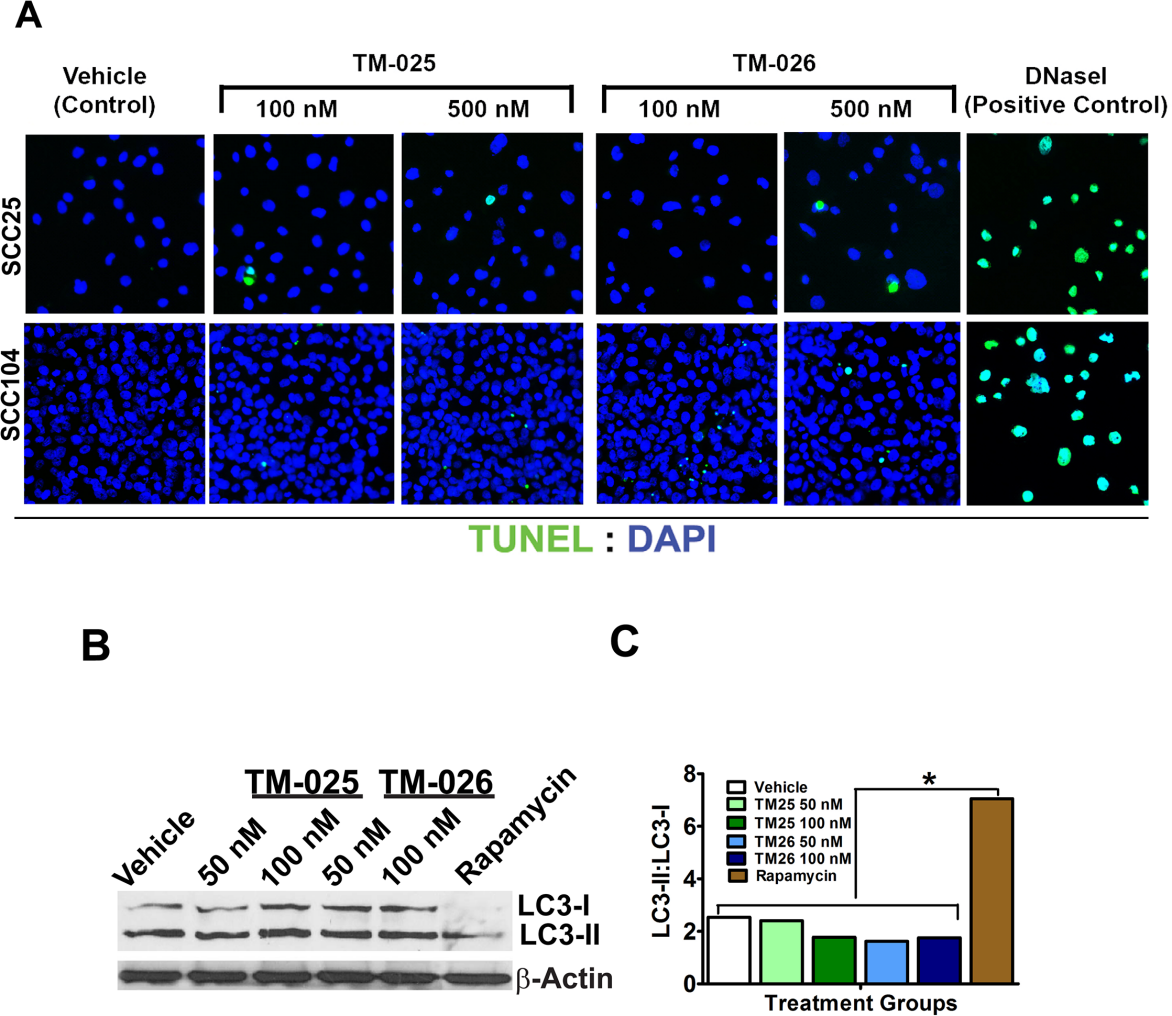
PCT analogs did not cause apoptosis or autophagy. (A): TUNEL assay: 24-h treatment with increasing concentrations (100 and 500 nM) of TM-025 and TM-026 did not show a significant (p<0.05) number of TUNEL^+^ apoptotic cells (green fluorescence) marked with arrow-heads in SCC25 and SCC104 cells. DNase I (100 ul; 10 U/ml; positive control) treated cells were used as a positive control. (B-C): Autophagy was estimated as a measure of ratio of cellular LC3-II to LC3-I. (B) Western blot depicting levels of LC3-I and LC3-II in PCT analog-treated cells. (C) The ratio of LC3-II:LC3-I did not show a significant (P<0.05) induction (represented by *) of autophagy in the PCT analog-treated cells, in comparison to the positive control (cells treated with 100 μl of 50 ng/ml rapamycin). All sample groups were found to be identical to the vehicle-treated group at the same level of significance (i.e., P<0.05).

In order to determine whether the PCT analogs induce cell death, besides inhibiting cell proliferation, both apoptosis and canonical autophagy assays were performed. Fluorometric TUNEL analysis with TM-025 and TM-026-treated SCC25 and SCC104 cells (100 and 500 nM; 24 h) did not reveal any significant percentage of TUNEL^+^ cells after 24 h of treatment, in comparison to the DNase I-treated positive control [51] (Fig 4A). Along the same line, western blot analyses of a relatively high dose (1 μM) of TM-025/TM-026-treated (for 72 h) SCC104 cells did not detect any cleavage of caspase-3 (S2 Fig Part A) or PARP (S2 Fig Part B), using specific antibodies against anti-cleaved caspase-3 and PARP, respectively. Hence, for the considered doses and treatment time, TM-025 and TM-026 did not induce apoptosis in either of the two HNSCC cell lines. Furthermore, occurrence of autophagy by TM-025 or TM-026 treatment in SCC104 cells was determined by detecting and comparing levels of LC3-I and LC3-II cytosolic proteins by Western blot analyses after drug treatment (Fig 4B). Rapamycin-treated cells were used as a positive control (Fig 4B). Neither of the PCT analogs induced any autophagy (up to 100 nM of treatment for 48 h), as illustrated by the LC3-II: LC3-I ratio (Fig 4C). Altogether, our results suggest that TM-025 and TM-026 do not reduce cell viability *via* apoptosis or autophagy.

### Induction of mild senescence in SCC cells when treated with PCT analogs

**Fig 5:**
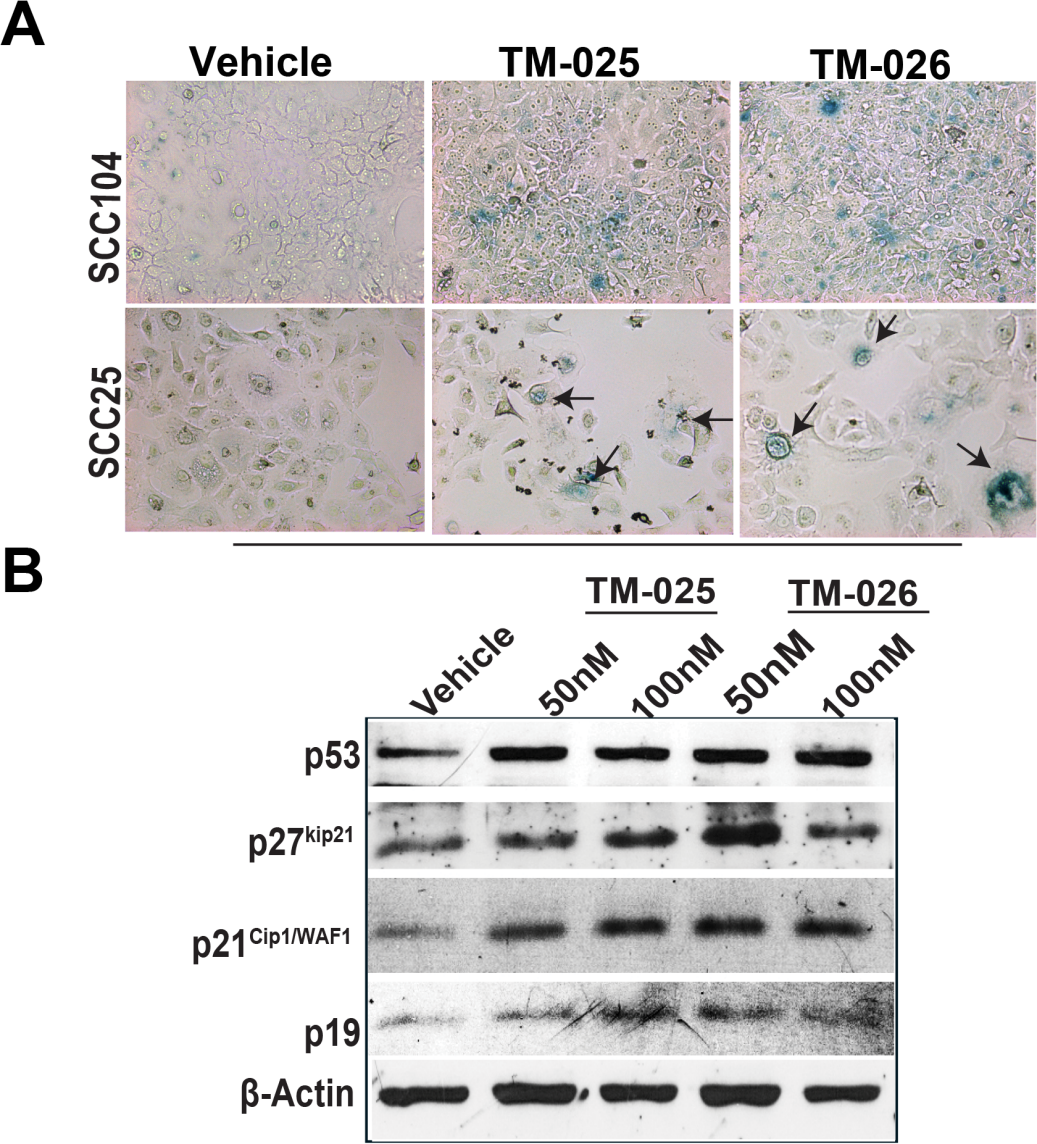
PCT analogs caused senescence in SCC25 and SCC104 cell lines by upregulation of p53, p21^Cip1/WAF1^ and p27^kip21^. (A): SCC25 and SCC104 cells were treated with DMSO (vehicle), 100 nM of TM-025 and TM-026 for 3 days, followed by SA-*β*-gal staining. A prominent onset of senescence (blue) was observed in both cell lines. (B): Western blots showing activation of p53, p21^Cip1/WAF1^ and p27^kip21^ by 50 and 100 nM of the PCT analogs. Quantifications are shown in S4 Fig Part A.

Cell cycle arrest is one of the key features of senescent cells *in vitro* and *in vivo* [52]. Also, a number of chemotherapeutic agents possess their anticancer activity by inducing senescence in the growth arrested cells [53]. We therefore investigated if the PCT analogs reduce cell viability by inducing senescence in SCC25 and SCC104 cells. Cells were treated with 100 nM of TM-025 and TM-026 for 72 h, followed by SA-*β*-gal staining in the two SCC cell lines (Fig 5A; for details see Methods). We observed significant (P<0.05) increase in number of SA-*β*-gal^+^ cells when treated with either TM-025 or TM-026 compared to the untreated/vehicle-treated controls in both SCC25 and SCC104 cell lines. Briefly, the number of SA-*β*-gal^+^ cells in TM-025 and TM-026 treated SCC104 cell line (TM-025: 12.5 ± 1.36; TM-026: 20.43 ± 2.57) and in SCC25 (TM-025: 14.65 ± 0.35; TM-026: 15.19 ± 0.19) were higher than the controls (SCC104 control: 6.00 ± 0.05 and SCC25 control: 3.4 ± 0.4). Prolonged treatment with PCT analogs (8 days) induced growth arrest and change in cell morphology and size. We observed large flat cells with increased volume. Some cells were also multinucleated, vacuolated and exhibited increased granularity (S3 Fig Scheme A) [54]. Many of these cells were SA-*β*-gal^+^ in case of both TM-025 and TM-026. On the contrary, vehicle-treated cells had reached maximum confluence at this time point and very few SA-*β*-gal^+^ cells were observed in such cases. The cells recovered partially when the plates were replenished with fresh medium without the drugs on day 4; but still retained the same large size with excess granularity. We observed a better recovery rate following withdrawal of TM-026 compared to TM-025, and the SA-*β*-gal^+^ cells were also low in case of the former. Interestingly, we continued to see large flat cells after withdrawal of TM-025 treatment and replacement with fresh medium on day 4, and observed increased number of SA-*β*-gal^+^ cells in these TM-025-withdrawn cells on the eighth day after SA-*β*-gal assay (S3 Fig Scheme B).

Activation of p53, p21^Cip1/WAF1^, p19 and p27^kip21^ are hallmarks of cells undergoing cell cycle arrest senescence [53, 55–57]. Therefore, we analyzed the expression of these proteins by western blot analysis after 72 h of treatment with PCT analogs. Immunoblotting demonstrated an induction of p53, and its downstream target p21^Cip1/WAF1^, along with p19 and p27^kip21^ proteins in cells treated with TM-025 and TM-026 for 72 h (Figs 5B and S4 Part A). Altogether, above results suggests that PCT analogs induce premature senescence in SCC25 and SCC104 cells.

### PCT analogs induce cell cycle arrest and drive expression of regulators of cell cycle progression

**Fig 6:**
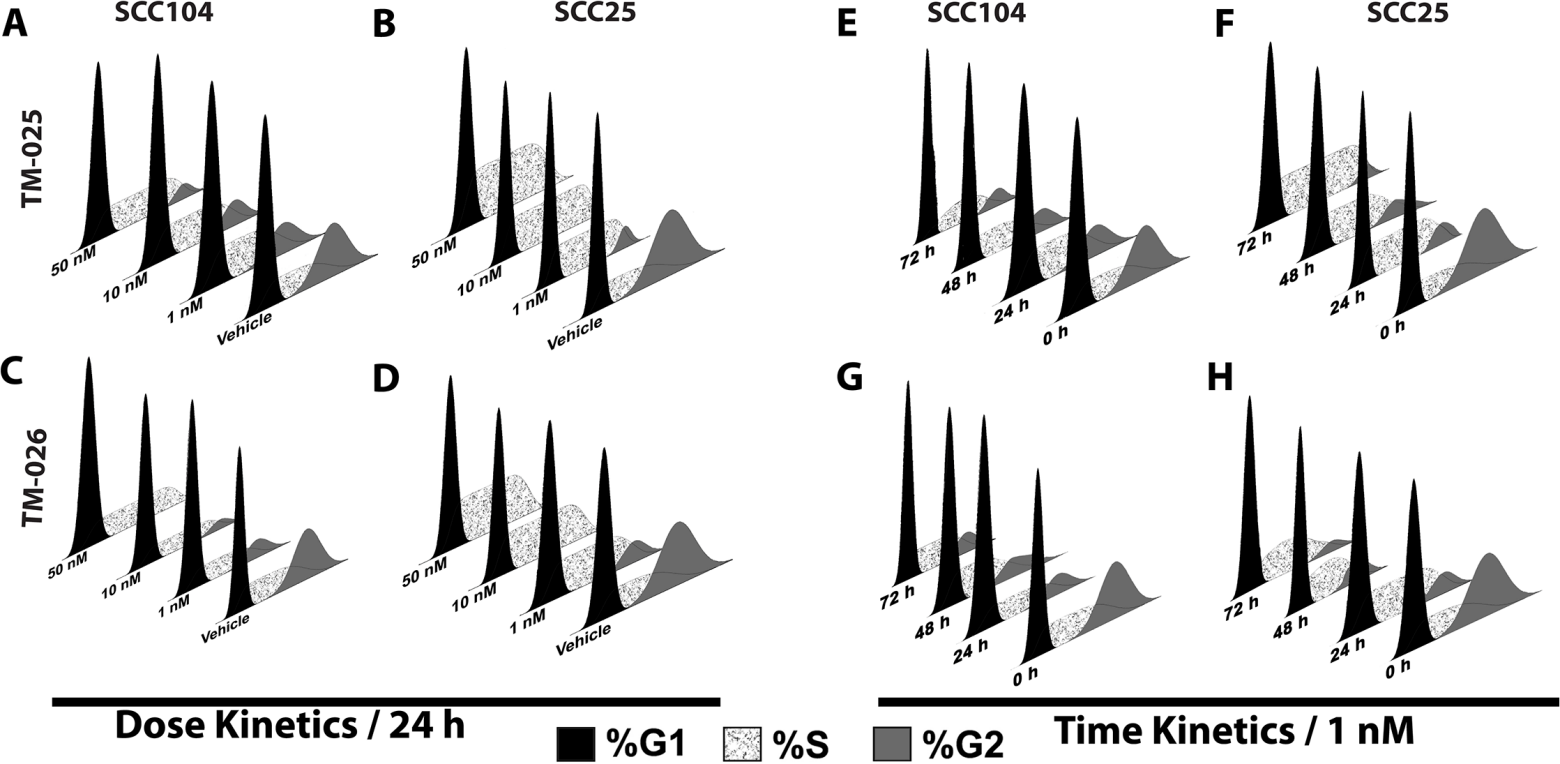
Treatment with PCT analogs altered cell cycle progression in HNSCC cells. Flow cytometry-based cell cycle analysis of SCC104 and SCC25 cells upon treatment with PCT analogs. Dose kinetics (A–D) was determined for a period of 24 h (with 1, 10 and 50 nM doses). A significant decline in the percentage of G_2_ cells was observed, along with a considerable augmentation in the percentage of S-phase cells. Time kinetics (E–H), on the other hand, was investigated for 24, 48 and 72 h for a dose of 1 nM. A time-dependent inhibition of cell cycle progression was observed. All results are significant at P<0.01.

We hypothesized that increased cytotoxicity, reduced cell viability, and reduced proliferation after treatment with PCT analogs could be due to inhibition of cell cycle progression. In order to investigate the effect of TM-025 and TM-026 on cellular distribution, we performed cell cycle analysis on SCC25 and SCC104 cells by flow cytometry, after staining the nuclei with propidium iodide, following treatment (1, 10 and 50 nM) with TM-025 or TM-026 for 24 h (for details see Methods). Respective percentages of G_1_, S and G_2_ phase-specific cells were evaluated and plotted (Fig 6). We observed an increase in S-phase from 17% in vehicle-treated to 36% and 27%, along with a concomitant decrease in G_2_-phase from 32% (vehicle treatment) to 15% and 12% in SCC104 cells, after 24 h of treatment with 1 nM of TM-025 and TM-026, respectively (Fig 6A and 6C and S1 Table). Similarly, an increase in S-phase from 17% (vehicle-treated) to 35% and 48% and a simultaneous decrease in G_2_-phase from 27% (vehicle treatment) to 12% and 2% for SCC25 cells were observed after a 24 h treatment with 1 nM of TM-025 and TM-026, respectively (see Fig 6B and 6D and S1 and S2 Tables). Strikingly, there was a complete loss of G_2_-phase cells when treated with higher concentrations (10 and 50 nM) of TM-025 and TM-026 in the SCC25 cell line (Fig 6B and 6D and S2 Table). Similarly, a time-dependent (24, 48 and 72 h) trend in S-phase cell-cycle arrest and a reduction or complete loss of G_2_-phase cells were observed in the SCC25 and SCC104 cells after treatment with TM-025 or TM-026 at a concentration of 1 nM (Fig 6E–6H and S1 Table).

**Fig 7:**
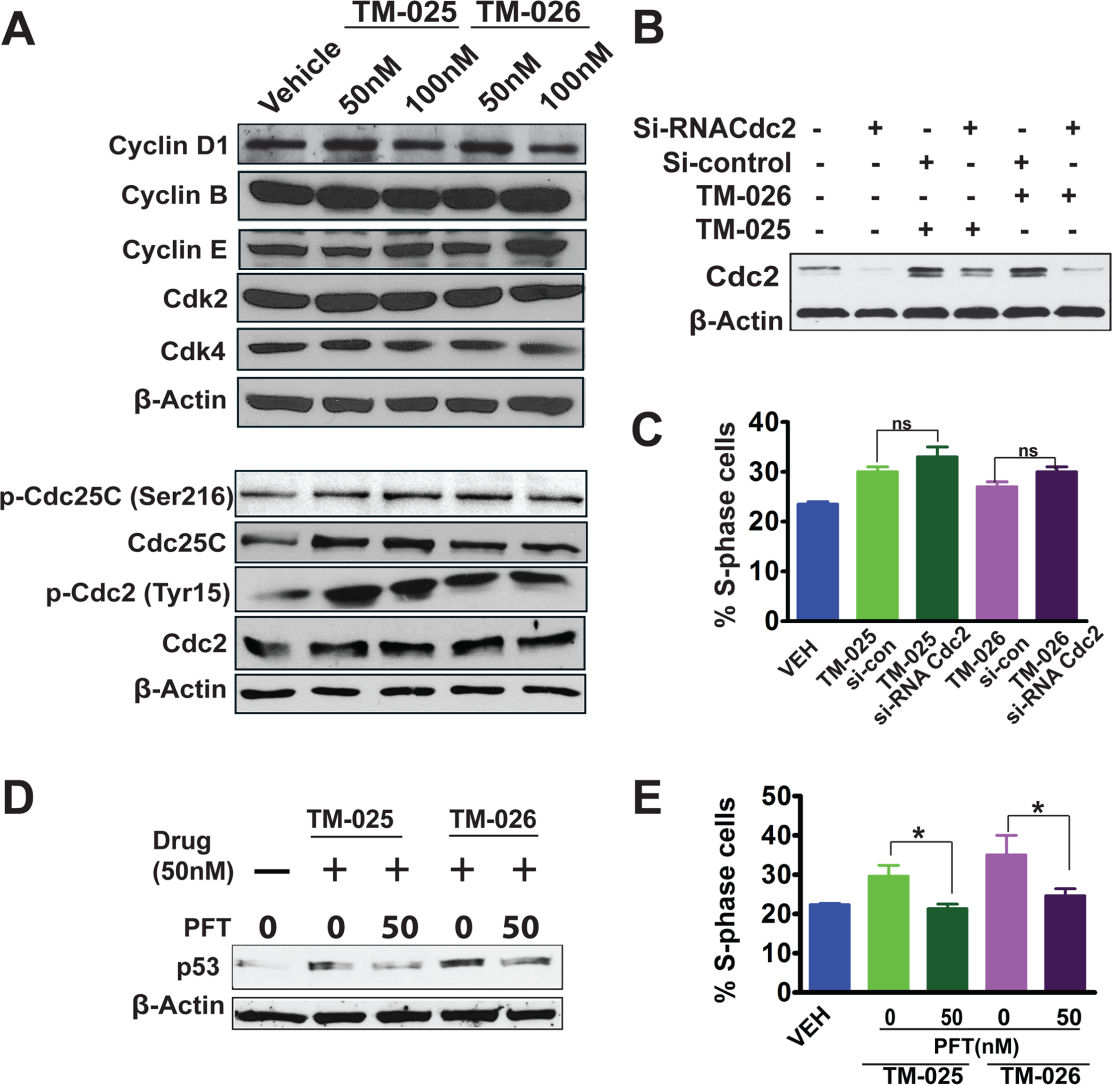
Altered expression of key cell cycle regulators, silencing of Cdc2 and inhibition of p53 in SCC25 cells by the PCT analogs. (A): Immunoblotting showed mild dose-dependent (50 and 100 nM) reduction in the expression of cyclin D1, moderate induction of cyclin E, and no or negligible effect on cyclin B. Both PCT analogs did not cause any change in the levels of Cdk2 and Cdk4, but induced the expression of Cdc2 (Cdk1) and Cdc25C. The analogs significantly augmented phosphorylation of Cdc2 at the Tyr15 residue, thus upregulating the level of phospho-Cdc2(Tyr15), which is an inactive form of Cdc2. Induction was also observed in the expression of phospho-Cdc25C(Ser216) in samples treated with TM-025/TM-026. Equal loading was confirmed by *β*-actin. Quantifications are shown in S4 Fig Part B. (B): siRNA mediated silencing of Cdc2. Cells were transfected with Si-Cdc2 and si-Con (control), and 24h later 50 nM of PCT analogs were added and incubated for 24h and harvested for protein extraction. Immunoblots showed knock down efficiency of the Cdc2 siRNA. Briefly, the gel showed untransfected cells, Cdc2 siRNA-transfected cells, Cdc2 siRNA or control siRNA-transfected cells treated with 50 nM PCT analogs. (C): Bar graph shows % S-phase cells after cell cycle analysis of propidium iodide (PI) stained cells. NS: not significant. (D): Pharmacological inhibition of p53 by PFT. Cells were treated with vehicle, PCT analogs, and PCT analogs with multiple doses of 50 nM PFT. Western blots showing inhibition of p53 by PFT at 72 h in TM-025 and TM-026 (50 nM) treated SCC25 cells. (E) Bar graph shows % S-phase cells after cell cycle analysis of PI-stained cells. Results showing significant changes are denoted by * (P<0.05) and not significant denoted as ns. β-Actin was used as loading control for A, B and D.

Since the PCT analogs induced cell cycle arrest in S-phase with a loss of G_2_-phase cells (Fig 6), we examined the effects of TM-025 and TM-026 on the levels of some of the cell cycle regulators, such as cyclins, cell division cycle (Cdc) proteins and cyclin-dependent kinases (CDKs) by Western blot analysis. Our results demonstrated a mild dose-dependent (50 and 100 nM of TM-025/TM-026) reduction of cyclin D1 and moderate induction of cyclin E, with no significant changes in the levels of cyclin B, Cdk2 and Cdk4 (Figs 7A and S4 Part B). We also observed considerable augmentation in expression of total Cdc25C, as well as in phosphorylated (Ser216) form of Cdc25C (Figs 7A and S4 Part B). Similarly, there was a modest increase in the total Cdc2 protein level and increased phosphorylation of phosphatase Cdc2 at Tyr-15 residue (Figs 7A and S4 Part B). Altogether, the above results suggest that both the HNSCC cell lines exhibited an S/G_2_ checkpoint arrest, following treatment with TM-025 or TM-026, as demonstrated by significant dose-dependent increase in the percentage of S-phase cells, along with a concomitant decrease in G_2_-phase cells.

In order to confirm the role of Cdc2 in mediating S-phase arrest by TM compounds, we silenced Cdc2 in SCC25 cells using specific siRNA and performed immunoblotting and cell cycle analysis. Immuno-blotting revealed an efficient knockdown of Cdc2 expression induced by TM-025 and TM-026 (Fig 7B). Surprisingly, Cdc2 silencing failed to abrogate TM-025/TM-026-induced S-phase arrest (Fig 7C).

p53 has been identified to play an important role in cell-cycle progression, and we have also observed its induction after treatment with PCT analogs. Therefore, to confirm the role of p53 in TM-025 and TM-026 induced S-phase arrest, we used selective p53 inhibitor PFT to block p53 expression and performed cell cycle analysis in SCC25 cells. Our western blot results showed reduction in TM-025 and TM-026 induced p53 expression, post PFT treatment (Fig 7D). Corresponding cell cycle analysis showed that addition of PFT was able to reverse the cell cycle block induced by the PCT analogs (Fig 7E). In particular, PFT treatment reduces S-phase from 30% to 21% in TM-025 treated cells and from 35% to 24% in TM-026 treated cells (Fig 7E). Altogether, the above results suggest that PCT analog-induced inhibition of cell cycle is mediated, at least in part, by p53 signaling and not by Cdc2 mediated signaling.

## Discussion

**Fig 8:**
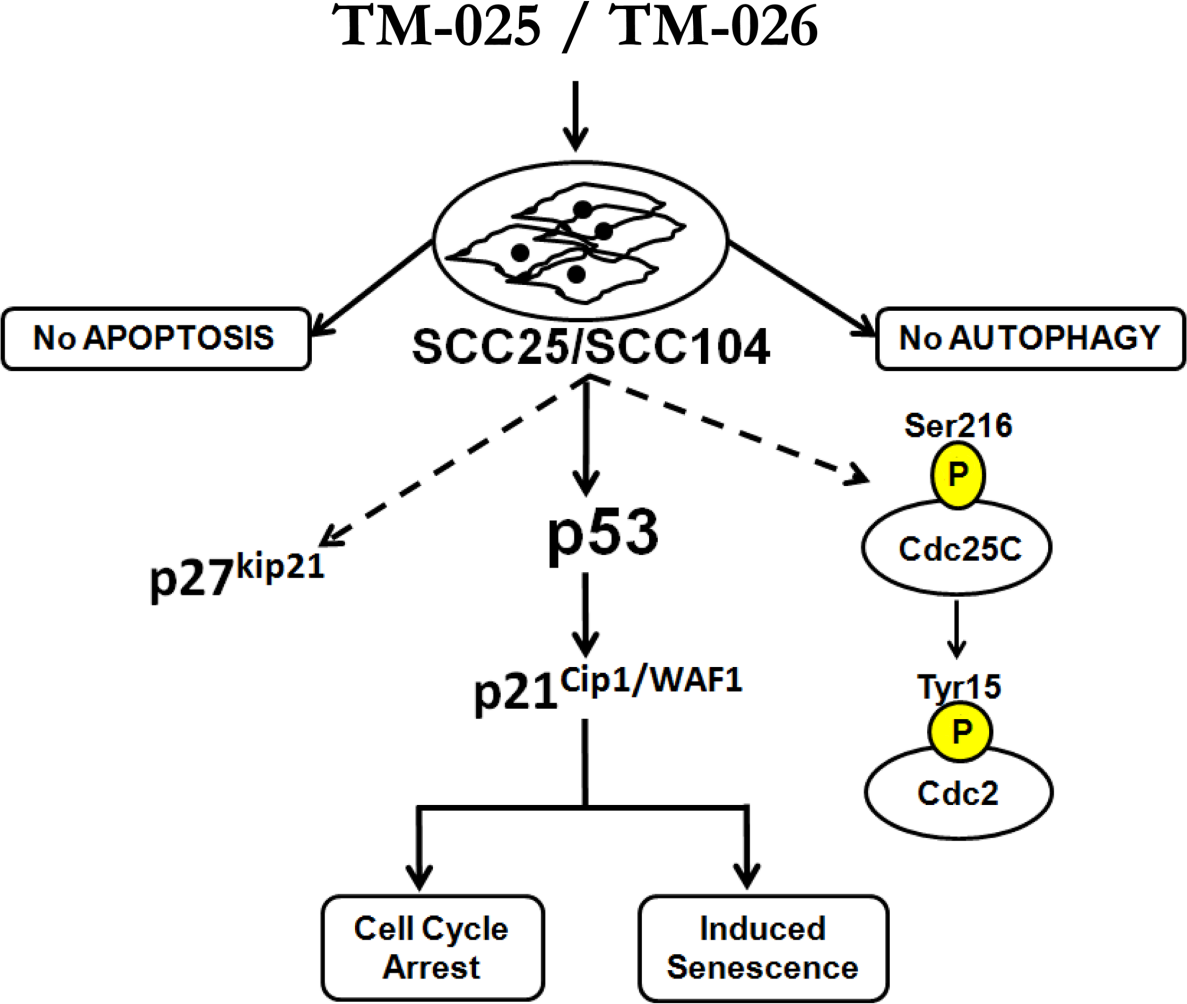
Schematic representation of mechanisms of action of the PCT analogs. Treatment of SCC25/SCC104 cells with TM-025 or TM-026 induced S-phase cell cycle arrest (S/G_2_-checkpoint) and senescence, but did not induce apoptosis or autophagy. At the molecular level, TM-025 and TM-026 induced expression of p53, its downstream target p21^Cip1/WAF1^, and also p27^kip21^, which are known to arrest cell cycle progression. Inhibition of p53 signaling by PFT abrogated TM-025/TM-026-induced S-phase arrest, thereby confirming a direct role of p53 in these processes. PCT analogs also enhanced phosphorylation of phosphatase Cdc25C (at Ser216 residue) and induced expression of total and Phsopho-Cdc2 (at Tyr15 residue). Inhibition of Cdc2 signaling by siRNA-mediated knockdown of Cdc2 did not relieve the drug-induced S-phase arrest. Solid arrows indicate confirmed mode of action of TM-025/TM-026 mediated *via* p53, while dotted arrows indicate additional downstream effectors of TM-025 and TM-026.

We have previously demonstrated that biosynthetically engineered PCT analogs (TM-025 and TM-026) are less toxic than the parent compound PCT towards mammalian cells [21]. In the present study, we demonstrated that the PCT analogs inhibit proliferation and induce senescence in HNSCC cell lines (SCC25 and SCC104). We have further elucidated their mechanisms of action in inhibiting viability of HNSCC cells. Our results confirmed that both TM-025 and TM-026 inhibit growth and reduce viability of cancer cells through possible contribution of the p53 signaling pathway, as well as inducing senescence in those cells (schematically described in Fig 8).

The effects of the two PCT analogs were tested in two established squamous cell carcinoma cell lines and in normal epithelial cells (HPEK) [58,59]. Overall, MTT-formazan quantification (MTT assay) showed significant dose- and time-dependent decline in the viability of all three cell lines following treatment with PCT analogs. Results indicated that TM-025 and TM-026 interfere with the propagation and growth of those cells at the S-phase and at a higher concentration induced G2-phase arrest at different timepoints (data not shown). To investigate the cellular mechanisms of TM-025 and TM-026 activity, we analyzed diverse cellular processes, including proliferation, apoptosis, autophagy and senescence. Results obtained from those different assays confirmed that both the PCT analogs inhibit proliferation of the SCC cells, as well as of normal human epithelial cells. Although there are some anticancer drugs which negligibly affect normal cells, most popular anticancer drugs such as 5-fluorouracil (5-FU) and cisplatin also reduce viability and induce apoptosis of normal cells as a collateral effect [60]. However, none of the tested compounds induced apoptosis in HNSCC cells, even after prolonged treatment (72 h) at a higher concentration (1 μM), as evaluated by TUNEL assay or by Western blot analyses for cleaved caspase-3 and PARP cleavage. Activated (cleaved) caspase-3 is a key executioner of apoptosis [61], while inactivated (cleaved) PARP renders a cell vulnerable to the apoptotic machinery [62]. Although, expression of p53 was upregulated following drug treatment, we did not observe any apoptosis, since p53-induced apoptosis occurs only when an apoptotic threshold is reached [63], which was probably not attained in those cells.

Expression of microtubule-associated protein light chain 3 (LC3), which is utilized to examine autophagy by detecting conversion of LC3-I to LC3-II, was analyzed in HNSCC cells after drug treatment. The LC3-II:LC3-I ratio, also known as the cytosolic LC3 ratio [64], was considered as a measure of autophagy. The LC3-II:LC3-I ratio did not reveal a significant induction of autophagy in the cancer cells treated with varying doses of the PCT analogs in comparison to rapamycin-treated cells serving as a positive control.

We observed a dose-dependent S/G_2_ arrest in progression of cell cycle after treatment with the PCT analogs. The cell cycle process and entry of cells into mitosis is controlled by Cdc2 kinase [65]. The critical regulatory step in activating Cdc2 during progression into mitosis appears to be dephosphorylation of cdc2 at Thr-14 and Tyr-15 [66]. Activation of Cdc2 *via* dephosphorylation by the protein phosphatase Cdc25C is a crucial step for cell cycle progression [67]. We observed that TM-025 and TM-026 modestly induced phosphorylation of the Ser216 residue of Cdc25C, thereby rendering Cdc25C inactive [68]. In congruence, an augmentation in the level of total and phospho-Cdc2 was observed, suggesting that Cdc2 signaling might be important to drive the cell cycle arrest. Other authors have obtained similar results previously by treating ovarian carcinoma (Ovcar-3) cells with resveratrol, which induces S-phase arrest *via* phosphorylation of Cdc2 [69]. A number of other compounds, such as vanadate, certain retinoids and zidovudine, have been previously reported to cause similar S-phase arrest in a variety of cell types [70–73]. siRNA-mediated silencing of Cdc2 in TM-025 and TM-026-treated HNSCC cells did not abrogate the cell cycle arrest at S-phase. Interestingly, Cdc2 knock-down modestly reduces the accumulation of cells at the G2-phase at higher concentration of the analogs (data not shown). It has been previously shown that the G1/S-phase transition is controlled by two parallel pathways, one being Cdk2 and the other Cdc2. Therefore, inhibition of Cdk2 or Cdc2 alone results in little effect on S-phase but will sensitize the system for inhibition of the other kinase [74]. The lack of any observable effects of Cdc2 silencing on S-phase cells might be due to the role of other regulatory proteins (e.g. p53), associated with the cycling of cells during the S-G2 transition, to mediate the effects of the PCT analogs (Fig 8).

The process of cell cycle is very tightly integrated and is a critical regulator of cell proliferation and growth and of cell division after DNA damage [75]. There are several key cell cycle regulators that are crucial for proper functioning of the cell cycle, such as cyclins, CDKs [76] and CDK inhibitors (CDKI) [77,78]. Cdk2 and Cdk4, in complexes with cyclins A and E, respectively, control the S-phase of the cell cycle [79]. Although their expressions were unaltered after drug treatment, expression of p21^Cip1/WAF1^, a crucial inhibitor of cell cycle progression and a downstream target of p53 [80], was induced after treatment with the PCT analogs. Similarly, expression of p27^kip21^, which has antagonistic effects on S/G_2_ cell cycle progression [81], was enhanced in the PCT analogs treated HNSCC cells. We also observed an increase in p19 expression after treatment with the PCT analogs. p19 overexpression has been reported to induce G1/S phase delay and apoptosis [82], which were not observed in the PCT treated cells, suggesting an indirect effect of the PCT analogs on p19 expression. The above results suggested that the S/G_2_ cell cycle arrest induced by the PCT analogs might be due to contribution of p53 mediated upregulation of p21^Cip1/WAF1^ and p27^kip21^ in those head and neck cancer cells. In the same line, we showed that PFT, a selective inhibitor of p53, reverses the cell cycle block induced by the PCT analogs and facilitates cell-cycle progression in a dose-dependent manner. Previous studies with PFT have established its role to protect different cells against-p53 dependent apoptosis induced by multitude of stimuli [83–87].

A recent study established that upregulation of p53 below its apoptotic threshold might drive the cells towards senescence [63]. Interestingly, we observed senescence in HNSCC cells post treatment with the PCT analogs for 72 h. Growth arrest of senescent cells is putatively initiated with the activation of p53 [53], followed by a canonical upregulation of p21^Cip1/WAF1^ [53,88]. p27^kip21^ also reportedly plays a role in causing cellular senescence [56,57]. PCT analogs might have followed a similar mode of action to induce senescence of HNSCC cells. In our long-term treatment study with higher dose of the PCT-analogs, we observed dramatic changes in cell morphology, such as large flattened cells with increased granularity and multiple nuclei similar to what is seen during mitotic catastrophe, in TM-025 treated cells both in the long term treatment group and after drug withdrawal, but not in the TM-026 treated cells. Those cells were also SA-*β*-gal^+^. This suggests that those cells might have undergone mitotic catastrophe at least in TM-025 treated cells, prior to undergoing senescence similar to what was reported earlier for LD doxorubicin-treated cells [89].

Overall, the present study provides insights into the mode of action of the PCT analogs in HNSCC cells, which includes (1) induction of Cdc2 and its phosphorylation *via* the Cdc25-pCdc2(Tyr15) pathway, (2) concerted upregulation of p53, p21^Cip1/WAF1^ and p27^kip21^, thereby contributing to cell cycle arrest at S/G_2_-phase and induction of cellular senescence (see Fig 8). We further demonstrated that these two compounds do not inhibit protein synthesis in general and, therefore, are unique in function when compared to PCT, the parent compound. Fig 8 illustrates the mode of action of TM-025 and TM-026 in SCC25 and SCC104 cell lines. There are number of different compounds which are known to induce cell cycle arrest at G_1_/S or G_2_/M-phases [90,91]. Many cytotoxic agents, which are used as chemotherapeutics, affect cell cycle phases in a very specific manner at precise stages, and have been widely used in the clinic as well as in basic research labs [92]. Elucidation of the biological aspects of tumor cell senescence offers plausible approaches for developing novel therapeutic strategies to stop the growth of tumor cells [53]. Hence, additional transcriptomic or proteomic analyses are necessary to identify those factors that directly mediate the anti-proliferative and cytotoxic effects of the newly characterized PCT analogs. The potential targets of these analogs in inhibiting cellular proliferation can also be identified *via* biotin conjugation of the PCT analogs followed by liquid chromatography–tandem mass spectrometry (LC-MS/MS) [93]. Analyses of the *in vivo* anti-tumoral effects of the two analogs might be useful to establish their therapeutic potential for prevention of HNSCC progression in humans. Given that the compounds are also active against normal cells, targeted drug delivery systems using nanoparticles loaded with the PCT analogs can be developed in the future.

## Supporting Information

S1 Fig PCT analogs reduced viability of human primary epidermal keratinocytes (HPEK) in a dose-dependent manner (MTT assay). 1×10^4^ cells of each type were treated with TM-025 and TM-026 for 24 h in increasing concentrations (1, 10, 100, 500, 1000 and 5000 nM). Percentage viability of HPEK cells were significantly (P<0.05) reduced with higher doses of both analogs. Results are plotted as means (±SEM).
(TIF)

S2 Fig PCT analogs did not induce apoptosis in HNSCC cells. (A) TM-025 and TM-026 did not induce caspase-3-mediated apoptosis. Immunoblot with an antibody specific against cleaved caspase-3 did not reveal band for cleaved caspase-3 following a high dose of PCT analog treatment (1 μM; 72 h) of SCC104 cells. (B) High dose (1 μM) of TM-025 or TM-026 treatment for 72 h did not reveal any PARP cleavage by Western blot analysis using anti-PARP antibody. *β*-actin was used as internal control.
(TIF)

S3 Fig Continuous treatment (8 days) with PCT analogs caused changes in cell morphology and induced senescence in SCC25 cell lines. Scheme A: Long-term continued treatment with 500 nM of TM-025 and TM-026 showed changes in cellular morphology and induction of SA-*β*-gal positive cells in SCC25 cells. Scheme B: Treatment with 500 nM of TM-025 and TM-026 for 4 days followed by drug withdrawal and continued culture for additional 4 days (Day 8) also demonstrated changes in cellular morphology. Induction of senescence and poor recovery rate were observed in TM-025-treated cells, while TM-026 treated cells recovered gradually and exhibited senescence. Arrows indicate large, flat, vacuolated SA-*β*-gal^+^ positive cells. Magnification: 20X.
(TIF)

S4 Fig Percentage changes in expressions of different cellular proteins with respect to vehicle control for 50 and 100 nM treatment with TM-025/26 (as observed by quantification of Western blots). (A): Quantification of Western blot (shown in Fig 5B): Expressions of p53, p27^kip21^, p21^cip1/WAF1^ and p19 were significantly increased (P<0.05) due to treatment with TM-025 and TM-026 in a dose-dependent pattern. (B): Quantification of Western blot (shown in Fig 7): Tested at 95% significance, cyclin D1 showed a mild dose-dependent reduction in expression, while cyclin E was moderately upregulated. Considerable and significant (P<0.05) upregulation was also observed in the expressions of Cdc2 (Cdk1), phospho-Cdc2 (Tyr15), Cdc25C and phospho-Cdc25C (Ser216). No significant changes were observed in the levels of cyclin B, Cdk2 and Cdk4. Results showing significant changes are denoted by *** (P<0.05), and those not showing any significant changes (P≮0.05) are denoted by the **#** symbol.
(TIF)

S1 Table Cell cycle analysis of TM-025 & TM-026 treated SCC25 and SCC104 cells at different time points. Effects of 1 nM concentrations of TM-025 & TM-026 in SCC25 and SCC104 cells for cell cycle analysis at 24, 48 and 72 h post-treatment.
(DOCX)

S2 Table Cell cycle analysis of TM-025 & TM-026 treated SCC25 and SCC104 cells at different concentrations. Effects of 1, 10 and 50 nM of TM-025 & TM-026 in SCC25 and SCC104 cells for cell cycle analysis at 24 h post-treatment.
(DOCX)
