# Supplementary figures and images for: Novel Pactamycin Analogs Induce p53 Dependent Cell-Cycle Arrest at S-Phase in Human Head and Neck Squamous Cell Carcinoma (HNSCC) Cells
Source: PLoS One. 2015 May 4;10(5):e0125322. doi: 10.1371/journal.pone.0125322 (PMC4418703; doi:10.1371/journal.pone.0125322)

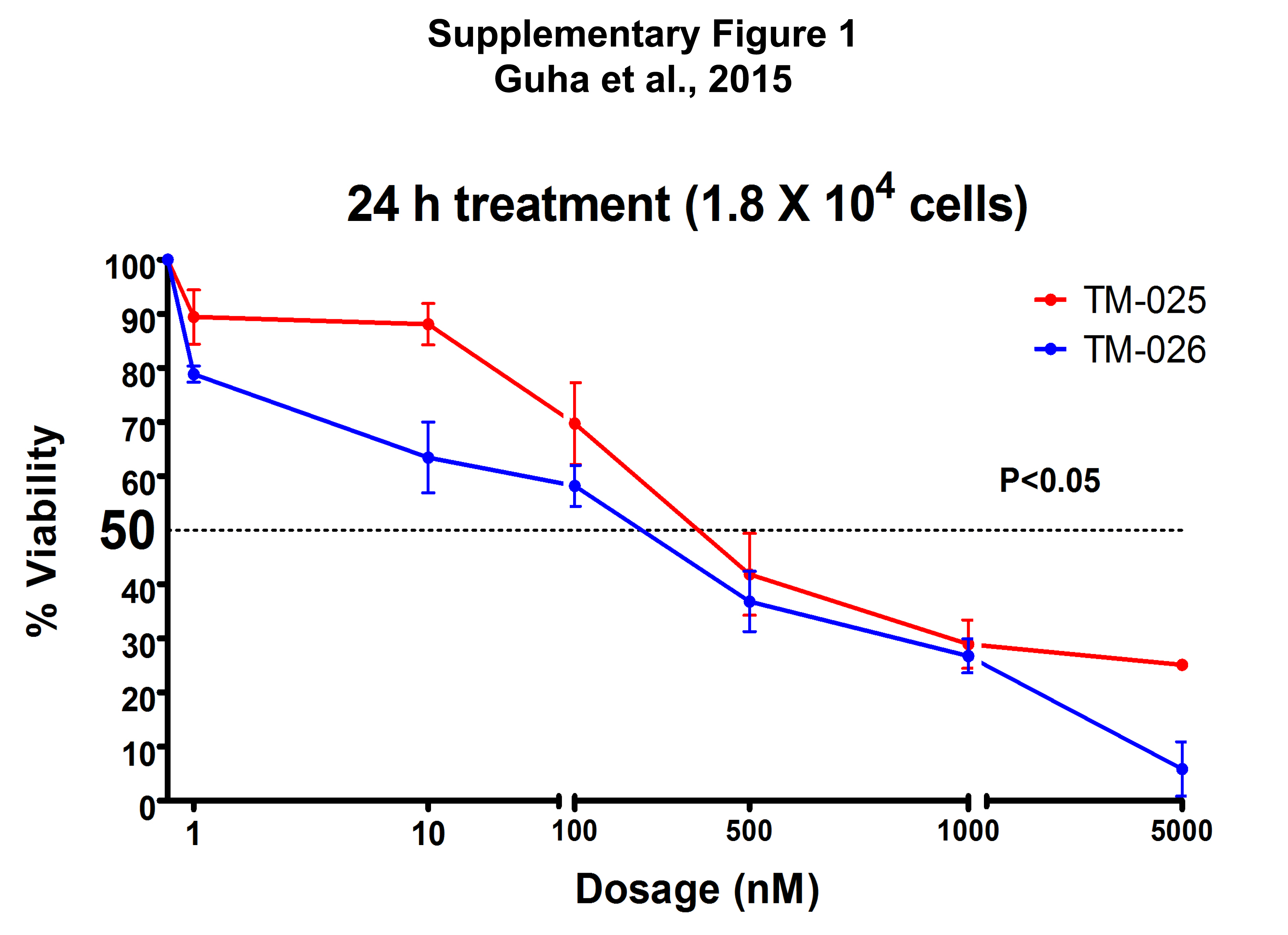

Supplement: S1 Fig — 1×10^4^ cells of each type were treated with TM-025 and TM-026 for 24 h in increasing concentrations (1, 10, 100, 500, 1000 and 5000 nM). Percentage viability of HPEK cells were significantly (P<0.05) reduced with higher doses of both analogs. Results are plotted as means (±SEM). (TIF) [file pone.0125322.s001.tif]

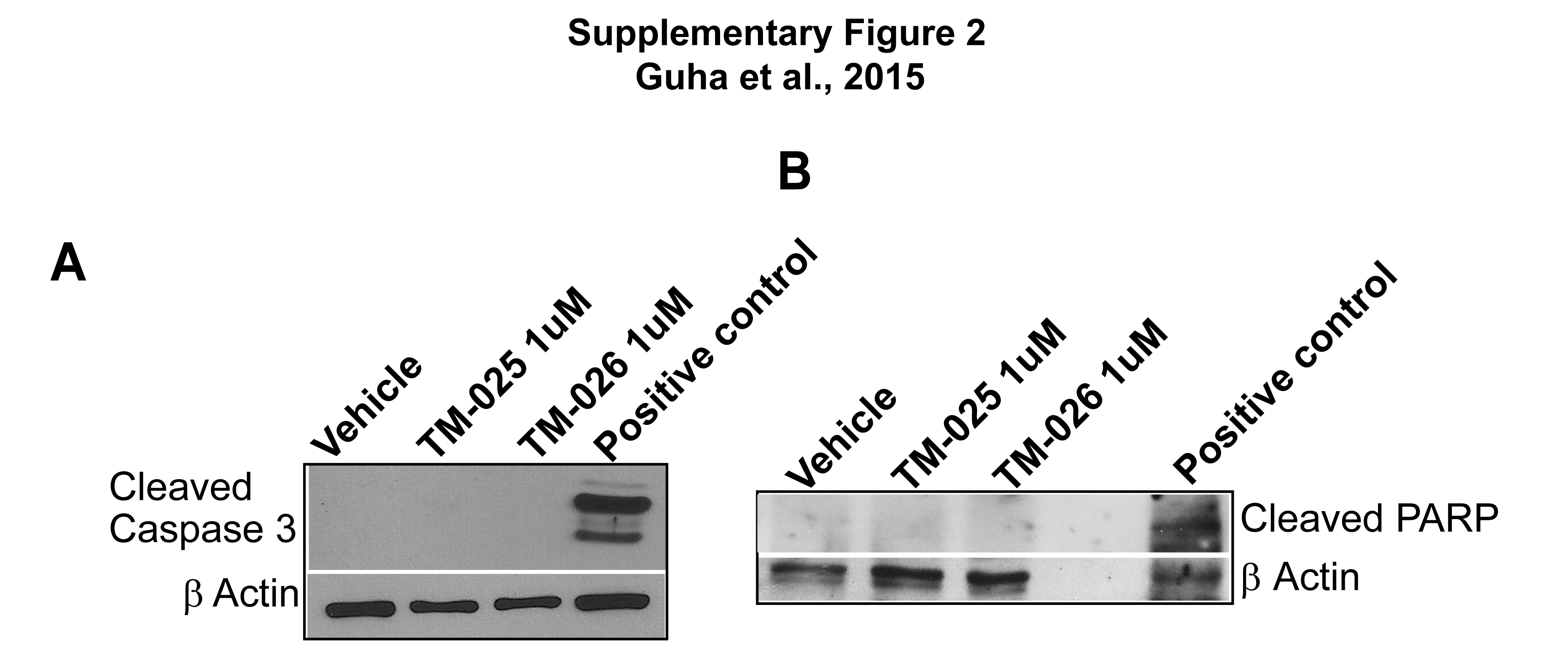

Supplement: S2 Fig — (A) TM-025 and TM-026 did not induce caspase-3-mediated apoptosis. Immunoblot with an antibody specific against cleaved caspase-3 did not reveal band for cleaved caspase-3 following a high dose of PCT analog treatment (1 μM; 72 h) of SCC104 cells. (B) High dose (1 μM) of TM-025 or TM-026 treatment for 72 h did not reveal any PARP cleavage by Western blot analysis using anti-PARP antibody. β-actin was used as internal control. (TIF) [file pone.0125322.s002.tif]

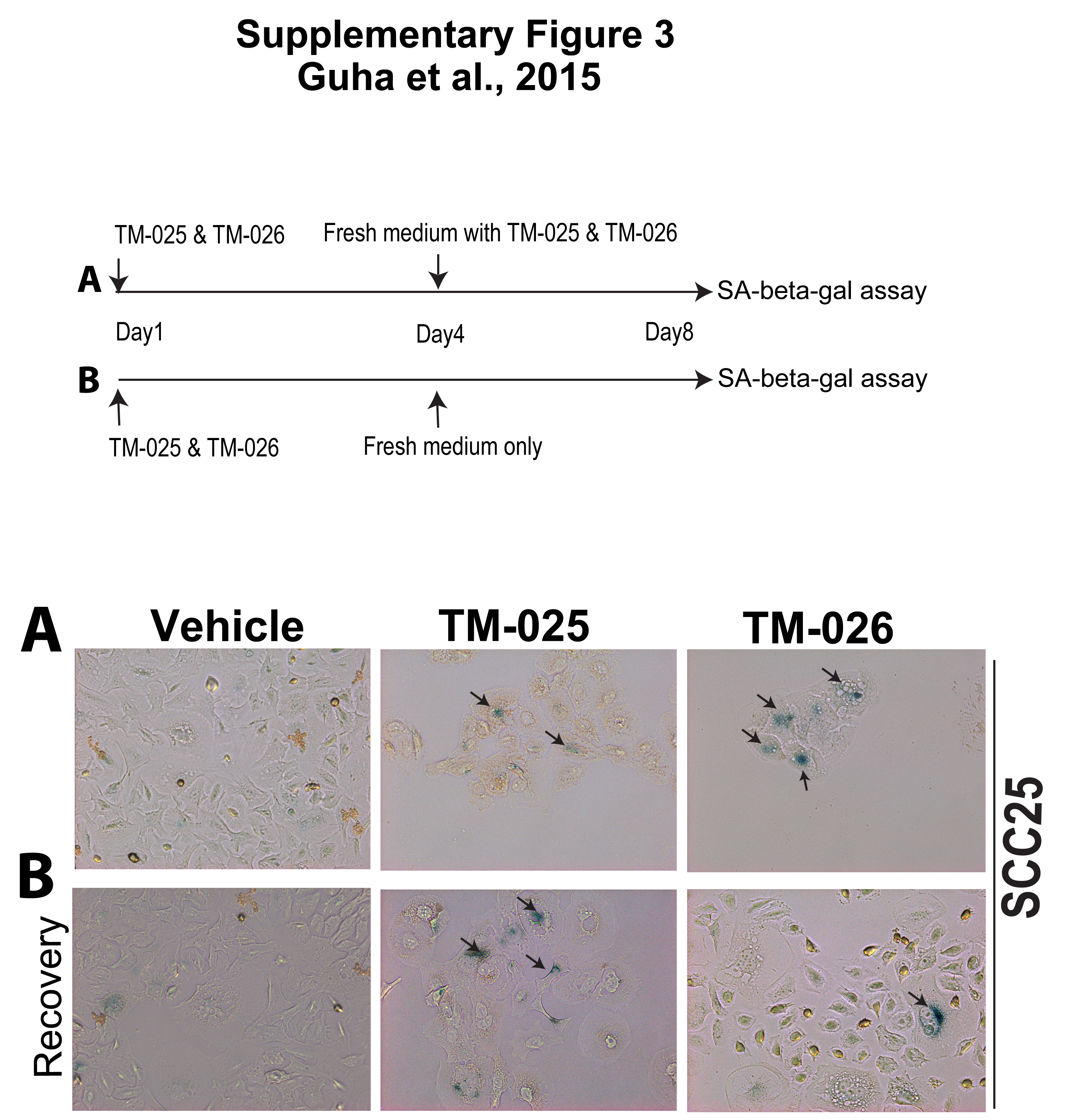

Supplement: S3 Fig — Scheme A: Long-term continued treatment with 500 nM of TM-025 and TM-026 showed changes in cellular morphology and induction of SA-β-gal positive cells in SCC25 cells. Scheme B: Treatment with 500 nM of TM-025 and TM-026 for 4 days followed by drug withdrawal and continued culture for additional 4 days (Day 8) also demonstrated changes in cellular morphology. Induction of senescence and poor recovery rate were observed in TM-025-treated cells, while TM-026 treated cells recovered gradually and exhibited senescence. Arrows indicate large, flat, vacuolated SA-β-gal+ positive cells. Magnification: 20X. (TIF) [file pone.0125322.s003.tif]

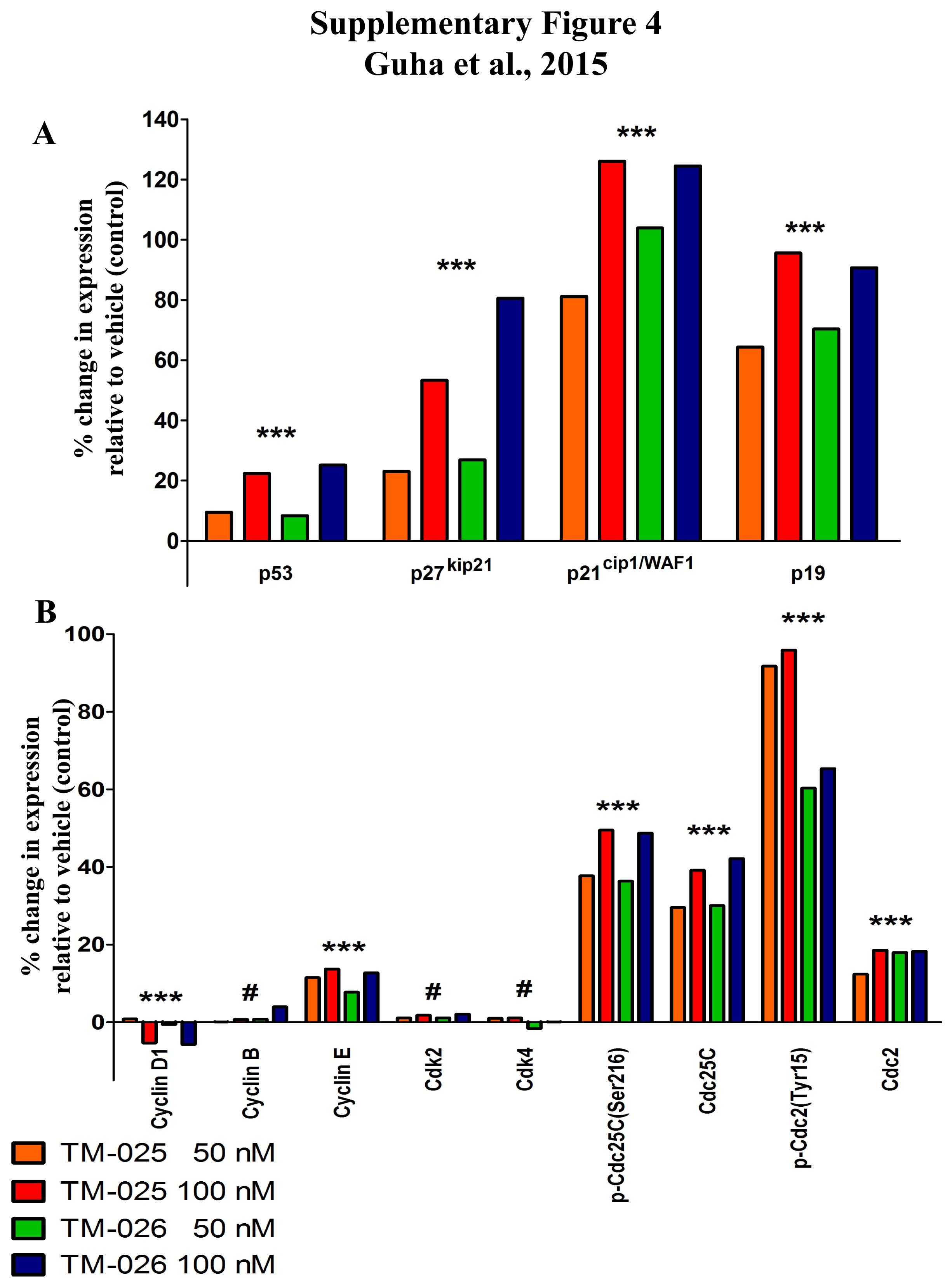

Supplement: S4 Fig — (A): Quantification of Western blot (shown in Fig 5B): Expressions of p53, p27kip21, p21cip1/WAF1 and p19 were significantly increased (P<0.05) due to treatment with TM-025 and TM-026 in a dose-dependent pattern. (B): Quantification of Western blot (shown in Fig 7): Tested at 95% significance, cyclin D1 showed a mild dose-dependent reduction in expression, while cyclin E was moderately upregulated. Considerable and significant (P<0.05) upregulation was also observed in the expressions of Cdc2 (Cdk1), phospho-Cdc2 (Tyr15), Cdc25C and phospho-Cdc25C (Ser216). No significant changes were observed in the levels of cyclin B, Cdk2 and Cdk4. Results showing significant changes are denoted by *** (P<0.05), and those not showing any significant changes (P≮0.05) are denoted by the # symbol. (TIF) [file pone.0125322.s004.tif]
