# Supplementary material for: Novel Pactamycin Analogs Induce p53 Dependent Cell-Cycle Arrest at S-Phase in Human Head and Neck Squamous Cell Carcinoma (HNSCC) Cells
Source: PLoS One. 2015 May 4;10(5):e0125322. doi: 10.1371/journal.pone.0125322 (PMC4418703; doi:10.1371/journal.pone.0125322)
Supplement: S1 Table — Effects of 1 nM concentrations of TM-025 & TM-026 in SCC25 and SCC104 cells for cell cycle analysis at 24, 48 and 72 h post-treatment. (DOCX) [file pone.0125322.s005.docx]

**S1 Table**

**Guha et al., 2015**

**S1 Table. Cell cycle analysis of TM-025 & TM-026 treated SCC25 and SCC104 cells at different time points.**

| **Treatment** |  | **SCC25** | | |  | **SCC104** | | |
| --- | --- | --- | --- | --- | --- | --- | --- | --- |
|  |  | **Vehicle** | **TM-025**  **(1 nM)** | **TM-026**  **(1 nM)** |  | **Vehicle** | **TM-025**  **(1 nM)** | **TM-026**  **(1 nM)** |
| **24 h** | **%G1** | 56.662 | 52.359 | 48.997 |  | 50.413 | 53.815 | 50.71 |
|  | **%S** | 16.548 | 35.224 | 48.205 |  | 16.844 | 46.185 | 48.692 |
|  | **%G2** | 26.789 | 12.418 | 2.818 |  | 32.743 | 15 | 12 |
|  | | | | |  |  | | |
| **48 h** | **%G1** | 56.662 | 60.341 | 60.231 |  | 50.413 | 53.854 | 60.694 |
|  | **%S** | 16.548 | 21.23 | 27.736 |  | 16.844 | 26.497 | 23.948 |
|  | **%G2** | 26.789 | 18.423 | 12.033 |  | 32.743 | 19.649 | 15.358 |
|  | | | | |  |  | | |
| **72h** | **%G1** | 56.662 | 52.551 | 60.875 |  | 50.413 | 53.588 | 55.236 |
|  | **%S** | 16.548 | 44.063 | 39.125 |  | 16.844 | 34.182 | 33.928 |
|  | **%G2** | 26.789 | 3.387 | 0 |  | 32.743 | 12.23 | 10.836 |
