# Supplementary material for: Novel Pactamycin Analogs Induce p53 Dependent Cell-Cycle Arrest at S-Phase in Human Head and Neck Squamous Cell Carcinoma (HNSCC) Cells
Source: PLoS One. 2015 May 4;10(5):e0125322. doi: 10.1371/journal.pone.0125322 (PMC4418703; doi:10.1371/journal.pone.0125322)
Supplement: S2 Table — Effects of 1, 10 and 50 nM of TM-025 & TM-026 in SCC25 and SCC104 cells for cell cycle analysis at 24 h post-treatment. (DOCX) [file pone.0125322.s006.docx]

**S2 Table**

**Guha et al., 2015**

**S2 Table. Cell cycle analysis of TM-025 & TM-026 treated SCC25 and SCC104 cells at different concentrations.**

| **Treatment** | **Cell Lines** |  | **Vehicle** | **TM-025** | | | **TM-026** | | |
| --- | --- | --- | --- | --- | --- | --- | --- | --- | --- |
|  |  |  |  | **1 nM** | **10 nM** | **50 nM** | **1 nM** | **10 nM** | **50 nM** |
| **24 h** | **SCC25** | **%G1** | 51.388 | 52.359 | 51.984 | 48.532 | 48.997 | 46.166 | 46.328 |
|  |  | **%S** | 17.529 | 35.224 | 48.016 | 51.468 | 48.205 | 53.834 | 53.672 |
|  |  | **%G2** | 31.083 | 12.418 | 0 | 0 | 2.818 | 0 | 0 |
|  |  |  |  |  |  |  |  |  |  |
|  | **SCC104** | **%G1** | 50.413 | 53.815 | 55.133 | 67.076 | 60.694 | 62.41 | 60.875 |
|  |  | **%S** | 16.844 | 26.497 | 28.499 | 24.084 | 23.948 | 31.529 | 39.125 |
|  |  | **%G2** | 32.743 | 19.649 | 16.368 | 8.840 | 15.358 | 6.061 | 0 |
